# Landscape configuration and habitat complexity shape arthropod assemblage in urban parks

**DOI:** 10.1038/s41598-020-73121-0

**Published:** 2020-09-29

**Authors:** Ming-Hsiao Peng, Yuan-Chen Hung, Kuan-Ling Liu, Kok-Boon Neoh

**Affiliations:** Department of Entomology, National Chung Hsing University, 145, Xingda Rd. South District, Taichung, 402 Taiwan, ROC

**Keywords:** Biodiversity, Urban ecology

## Abstract

The urbanization process systematically leads to the loss of biodiversity. Only certain arthropods are resilient to the urbanization process and can thrive in the novel conditions of urbanized landscapes. However, the degree to which arthropod communities survive in urban habitats depends on landscape and local effects and biological interactions (e.g., trophic interactions). In the present study, we examined the relative importance of various factors at landscape (isolation, edge density and area of surrounding greenery) and local (size of park, canopy cover, understory vegetation cover, defoliation depth, weight of dried leaves, soil temperature, soil moisture, and soil pH) spatial scales on the diversity of ants, beetles and spiders in urban parks. Our results indicated that park edge density was negatively correlated with diversity metrics in ants, beetles, and spiders in urban parks relative to the degree of proximity with the peri-urban forest. In other words, parks that located adjacent to the peri-urban forest may not necessarily have high biodiversity. The results suggested that man-made structures have been effective dispersal barriers that limit the spillover effects of ants and spiders but not the spillover of comparatively strong fliers, such as beetles. However, the area of surrounding greenery may have facilitated the colonization of forest-dependent taxa in distant parks. Large parks with reduced edge density supported a higher arthropod diversity because of the minimal edge effect and increased habitat heterogeneity. Vegetation structure consistently explained the variability of ants, beetles, and spiders, indicating that understory plant litter is crucial for providing shelters and hibernation, oviposition, and foraging sites for the major taxa in urban parks. Therefore, efforts should focus on the local management of ground features to maximize the conservation of biological control in urban landscapes.

## Introduction

Urbanization is spreading at an unprecedented rate in Asia, and the intense land transformation is a critical process contributing to the loss of biodiversity in the biodiverse tropics. Furthermore, urban expansion exaggerates urban heat island effects, causing temperatures in urban areas to be up to 10 °C–12 °C higher than those in the surrounding rural areas^1,2^. Therefore, urban habitats, with their impervious asphalt roads and buildings, are often considered disturbed habitats, hostile to arthropod survival^3^.

Urban landscapes are mosaics of man-made structure interspersed by urban greeneries of varying sizes that share varying degrees of connectedness to peri-urban forests. Intrinsic traits of participating organisms (e.g., phenotype) and extrinsic characteristics of the environment such as landscape configuration and local habitat heterogeneity are crucial in shaping local biodiversity and the associated ecological interactions in urban settings^4^. Studies have indicated that certain arthropods are resilient to the urbanization process, in which the changing environment evolutionarily selects organisms with life-history traits that enable survival in urban habitats^5–12^. However, the degree to which arthropod communities survive in urban habitats is dependent on landscapes and local effects. For instance, at the landscape scale, the spillover effect caused by the movement of a species to a low-quality habitat occurs through the influx of propagules from a source population inhabiting an adjacent high-quality habitat^13^, which may restore the loss of biodiversity in urban living spaces. Synergistically, landscape‐scale low-contrast habitat patch that characteristic of increased species richness and abundance inside habitat patches may reduce the impact of the isolation between urban parks and peri-urban forests^14,15^. These habitat patches may intensify inter-patch movement as well as provide additional or alternative foraging and nesting resources^16^. Spillover might also be a function of the habitat suitability surrounding patches. Fine-scale heterogeneity and structurally complex microhabitats may provide more niches spatially or temporally and allow organisms to utilize diverse methods to exploit environmental resources at a local scale^17,18^. Particularly in urbanized landscapes, habitat heterogeneity increases the survival potential of environment-sensitive species.

The arthropod assembly in urbanized landscapes and the diversities of prey and predators have been the focus of numerous studies on urban ecosystems^13,18–21^. The urbanization at both landscape and local spatial scales generally negatively affect the diversity of predator and parasitoid insects. For instance, the streets and buildings negatively affected the diversity of predators (Ampulicidae, Sphecidae, and Crabronidae) and parasitoids (Tachinidae) in Rome, Italy, because these man-made structures limited the dispersal capacity of these predators and parasitoids^22^. Similarly, local effects (garden size, mulch cover, the height of herbaceous vegetation, and tree and shrub richness) and landscape characteristics of the urban cover within 500 m of gardens affected parasitoid diversity in gardens in three counties of the California central coast: Santa Cruz, Santa Clara, and Monterey^20^. Notably, most predators and parasitoids that survive in urbanized landscapes are habitat generalist species and are thus tolerant to the urbanization process^19,22^.

Ants and beetles comprise a hyperdiverse group of insects and are crucial in an ecosystem^23^ because of their different nesting and feeding guilds^24–28^. Spiders act as natural enemies and serve as predators’ food in numerous ecosystems^29–32^. Approximately 400–800 tons of prey are killed by global spider community every year^33^. Efforts to enhance predators’ and parasitoids’ diversity and their associated biological control services have been extensively investigated in high-value seminatural agroecosystems. Considering the increasing importance of biological conservation in urbanized landscapes and the escalating insect pest status in urban areas^34,35^, urban developments have prompted broader mandates that include biodiversity conservation and promotion of ecosystem services for biological control in cities. In the present study, we examined the effects of landscape and local scale on the diversity of three major taxa in urban parks in Taichung city, Central Taiwan. We hypothesized that arthropod richness and diversity metrics in urban parks increase with proximity to peri-urban forests. However, this arthropod diversity responds variably to the effects of landscape configuration, composition of habitat, and local habitat heterogeneity depending on their survival potential and life-history traits.

## Materials and methods

### Study site

The study sites were located in Taichung city (24°04′–24°21′ N, 120°35′–120°41′ E), the second most populated city in Taiwan (approximately 2.8 million people). Taichung experiences a subtropical climate with a mean annual temperature of 23.3 °C, annual rainfall of 1712.1 mm, and relative humidity ranging from 72.3% (December) to 77.9% (June; according to Taiwan central weather bureau). The urban landscape is a mosaic of urban parks, median, agricultural fields, and residential houses. The study area was restricted to urban parks to reduce differential habitat characteristics that may cause difficulty in interpreting the hypothesized ecological processes in the study. In general, urban parks had an intermediate level of disturbance: these sites were usually dominated by grass, herbaceous plants, and recreational structures. Activities such as lawn mowing are prevalent here. A total of 47 parks (size from 0.16 to 27 ha) were selected for sampling. Each park was sampled once between July and October 2016. The parks were at least 1 km apart to ensure each data set obtained was independent.

### Sampling and specimen identification

This study aimed to assess the relationships between arthropod assemblages from three major taxa (i.e., ants, beetles, and spiders) and landscape metrics, and habitat complexity in urban areas. Ants, beetles, and spiders were collected using a standardized pitfall trap. The pitfall trap consisted of plastic container (diameter, 12 cm; height, 9 cm) containing a solution of ethylene glycol and water at a ratio of 1:2. Each pitfall trap was shielded by a corrugated plastic board (16 × 16 cm) to prevent it from getting filled with rain. Based on Liu, et al.^12^, three to seven pitfall traps were set up for four days in each park at three survey points: a group of trees, an isolated tree, and shrubs. The number of the pitfall trap set in the park was dependent on the size of park. The study regarding the locations indicated that this methodology sampled numerous ground-dwelling ants and achieved up to 80% sampling completeness^12^. The specimens were transported to the laboratory for further sorting. The specimens were preserved in 90% alcohol until they were identified. A data set of ant published by Liu, et al.^12^ was used. Yet, the ant species were resorted into genera based on identification keys for the ant fauna of Taiwan to reduce their influence of singletons and doubletons on the final result^36,37^. Beetle and spider specimens were assigned to the family level because sorting the specimens to a higher taxonomic resolution was not possible given the numerous specimens and inadequate appropriate keys. The identifications were based on Johnson and Triplehorn^38^, and assisted by spider taxonomists in Department of Life Science, Tunghai University. Timms, et al.^39^ reported that coarser levels of taxonomic resolution of beetle and spider do not significantly alter the distribution patterns in beta diversity and composition structure compared with higher taxonomic resolutions of the genus or species level. Studies have demonstrated congruence between family-level and species-level taxonomic resolution on the patterns of diversity metrics and compositional structure of our test taxa across treatment effects^39,40^; however, other reports have documented otherwise^8,41,42^.

### Landscape configuration

The landscape configurations that were considered in the study were isolation, edge density and area of surrounding greenery. Isolation refers herein to the tendency for survey parks to be relatively isolated in space from the peri-urban forests. The location of the urban park and nearest peri-urban forest were identified, and the distance from the peri-urban forest boundary was determined. The area of surrounding greenery (i.e., cover of arable fields and mixed semi-natural vegetation) at a scale of 2-km radius from each park was measured (the distance was based on the maximum flight distant of beetle^43^). All measurements were made using QGIS version 3.12.3^44^. The edge density was calculated as total perimeter of park (m) / park size (m^2^). The size of each survey urban park was obtained from Taichung city hall (https://opendata.taichung.gov.tw/dataset/3ac4d845-1a9f-11e8-8f43-00155d021202).

### Environmental variable measurements

The in situ environmental variables of each survey point were measured for a 10-m radius from where the pitfall trap was placed to assess the structural complexity. These environmental variables were canopy cover, defoliation depth, and soil profiles, which included surface temperature, moisture, and pH. Moreover, a 1-m^2^ quadrat was set at each survey point to estimate the percentage of understory vegetation cover. The plant litter within the quadrat was transported back to the laboratory to assess the dried weight.

### Data analyses

We calculated the richness and activity density of ant, beetle, and spider taxa for each survey park. The subsequent analysis was twofold. First, multiple linear regressions were then used to examine whether landscape-scale effects such as isolation, edge density and area of surrounding greenery significantly predict the arthropod assemblage in each survey park. Second, the local-scale effects of whether in situ environmental variables explained arthropod assemblages in each survey park were also examined. All models’ performances were evaluated using the Akaike information criterion (AIC). Furthermore, two measures associated with AIC, namely ∆AICc and AICc, were employed. The models were selected using package MuMIn version 1.40.4^45^ in R version 3.4.1^46^.

In addition, the association between community composition and in-situ environmental variables were analyzed (CANOCO version 5.0, Microcomputer Power, Ithaca, NY, USA). First, we determined the gradient length of the data set using detrended correspondence analysis. Since the gradient length was more than 4.0, the community composition occurred in each survey park was correlated with environmental variables using canonical correlation analysis (CCA) or redundancy analysis (RDA) was used otherwise. A Monte-Carlo test with 999 unrestricted permutations was conducted to test the significance of the environmental factors. The relative importance of environmental factor was represented by the length of arrow.

The analyses of the similarities of arthropod composition across parks of varying sizes were performed (PRIMER version 7, PRIMER-E Ltd., Lutton, UK). The data were first square-root transformed to reduce the variance of skewed activity density data. The parks were categorized into four groups according to size (Large park: > 4 hectare, n = 14; median park: 3.0–3.9 hectare, n = 6; small park: 2.0–2.9 hectare, n = 8; extra small park: < 2 hectare, n = 19) and seven categories of distance from the nearest forest (i.e., < 1.0 km, 1.0–1.9 km, 2.0–2.9 km, 3.0–3.9 km, 4.0–4.9 km, 5.0–5.9 km, and > 6.0 km). The species sampled were pooled across parks belonging to certain size and distance categories. Pairwise similarity matrices of arthropod composition are presented in a clustering dendrogram and heat map. The clusters were further analyzed using a similarity profile permutation test (SIMPROF).

## Results

We collected 13 343 ants from 22 genera. The genus *Monomorium* was omnipresent across the range of park sizes (100% occurrence), followed by *Pheidole* (98% occurrence) and *Tetramorium* (91% occurrence), whereas *Plagiolepis*, *Formica*, *Cerapachys*, *Anochetus*, *Recurvidris*, *Strumigenys*, *Anoplolepis*, *Polyrhachis,* and *Camponotus* were the rarest genera and was only found in one park. 464 beetles from 14 families were collected. Among the families, Anobiidae (38% occurrence), Lymexylidae (32% occurrence), and Carabidae (34% occurrence) were the most prominent. We collected 1245 spiders from 11 families. Salticidae was the most dominant in urban parks, comprising 91% of spider occurrence in the surveyed parks, whereas Thomisidae (2%) and Zodariidae (2%) were singletons.

### Association between arthropod diversity and landscape configuration

When isolation, edge density and area of surrounding greenery were combined in a multiple linear regression analysis, model selection by AICc indicated that edge density was the best model, with a model weight of 0.309, for ant genus richness, followed by edge density + area of surrounding greenery (model weight: 0.128) with a ΔAICc < 1.77 (Table 1). For ant activity density, edge density (model weight: 0.254) entered the model first, followed by edge density + area of surrounding greenery, isolation + edge density, and isolation + edge density + area of surrounding greenery (model weight: 0.215, 0.130, and 0.110, respectively) with a ΔAICc < 1.86.

**Table 1:** Results of the multivariate regression analysis testing the responses of the diversity metrics of three taxa to landscape configuration in study sites.

Response variable	Predictors	AICc	ΔAICc	Log likelihood	Akaike weight
**Ant**					
Richness	ED	192.0	0.00	− 92.741	0.309
	ED + GR	193.8	1.77	− 92.428	0.128
Activity density	ED	628.4	0.00	− 310.899	0.254
	ED + GR	628.7	0.34	− 309.870	0.215
	ISO + ED	629.7	1.34	− 310.372	0.130
	ISO + ED + GR	630.0	1.86	− 309.287	0.110
**Beetle**					
Richness	ED	180.7	0.00	− 87.095	0.528
	ISO + ED	182.4	1.68	− 86.740	0.227
Activity density	ED	296.8	0.00	− 145.126	0.306
	ISO + ED	297.0	0.18	− 144.017	0.280
	ED + GR	298.5	1.66	− 144.760	0.133
**Spider**					
Richness	GR	153.5	1.91	− 73.457	0.153
	ED	153.5	1.93	− 73.469	0.151
Activity density	ED	403.3	0.00	− 198.354	0.255
	ED + GR	403.3	0.04	− 197.176	0.250

Models are ranked according to model AICc. Only models with ∆AICc < 2 are displayed.

Abbreviations for landscape configuration: *ISO* isolation; *ED* edge density; *GR* area of surrounding greenery.

For beetle family richness, the landscape effect model with the best goodness of fit based on AICc was edge density (model weight: 0.528), followed by isolation + edge density (model weight: 0.227) with a ΔAICc < 1.68. For beetle activity density, the most important models (ΔAICc < 1.66) were edge density, isolation + edge density, and edge density + area of surrounding greenery, with model weights of 0.306, 0.280 and 0.133 respectively.

In the case of spider family richness, two models that performed the best (ΔAICc < 1.93) were area of surrounding greenery and edge density (model weight: 0.153 and 0.151, respectively). The model with the greatest goodness of fit for spider activity density included edge density (model weight: 0.255), edge density + area of surrounding greenery (model weight: 0.250), and area of surrounding greenery (model weight: 0.103) with a ΔAICc < 1.82.

### Association between arthropod diversity and environmental variables

Model selection to assess the ant genus richness produced four model candidates with a ΔAICc ≤ 1.87 (Table 2). The size was the best model predictor with a model weight of 0.187, followed by weight of dried leaves (model weight: 0.179), canopy cover (model weight: 0.120) and defoliation depth (model weight: 0.074). All the environmental factors were well fitted in the models for estimating ant activity density (ΔAICc ≤ 1.97), excluding temperature and defoliation depth. Size, canopy cover and understory vegetation cover displayed model weights of 0.209, 0.154, and 0.110 respectively.

**Table 2:** Results of the multivariate regression analysis testing the responses of the diversity metrics of three taxa to environmental variables in urban parks.

Response variables	Predictors	AICc	ΔAICc	Log likelihood	Akaike weight
**Ant**					
Richness	Size	192.6	0.01	− 93.026	0.187
	Weight of dried leaves	192.7	0.10	− 93.072	0.179
	Canopy cover	193.5	0.91	− 93.473	0.120
	Defoliation depth	194.5	1.87	− 93.957	0.074
Activity density	Size	629.8	0.00	− 311.610	0.209
	Canopy cover	630.4	0.61	− 311.913	0.154
	Understory vegetation cover	631.1	1.29	− 312.253	0.110
	Soil moisture	631.4	1.66	− 312.441	0.091
	Soil pH	631.6	1.81	− 312.516	0.085
	Weight of dried leaves	631.7	1.97	− 312.594	0.078
**Beetle**					
Richness	Understory vegetation cover	189.7	0.90	− 91.569	0.144
	Size	190.3	1.47	− 91.852	0.108
	Soil pH	190.4	1.61	− 91.923	0.101
	Soil temperature	190.6	1.78	− 92.009	0.093
	Canopy cover	190.7	1.86	− 92.049	0.089
	Defoliation depth	190.7	1.88	− 92.057	0.088
Activity density	Understory vegetation cover	301.2	1.81	− 147.319	0.103
	Soil pH	301.2	1.82	− 147.322	0.103
	Size	301.3	1.94	− 147.382	0.097
	Defoliation depth	301.3	1.94	− 147.384	0.097
**Spider**					
Richness	Weight of dried leaves	151.5	0.00	− 72.489	0.206
	Soil pH	152.0	0.44	− 72.709	0.166
	Size	153.4	1.89	− 73.436	0.080
Activity density	Soil pH	401.1	0.00	− 197.290	0.374
	Soil moisture	401.2	0.09	− 197.334	0.358

Models are ranked according to model AICc. Only models with ∆AICc < 2 are displayed.

We also constructed six model candidates for beetles with ΔAICc ≤ 1.88 produced from the model selection on beetle family richness prediction. Among model predictors, understory vegetation cover was the best model predictor with a model weight of 0.144, followed by size (model weight: 0.108), soil pH (model weight: 0.101), soil temperature (model weight: 0.093), canopy cover (model weight: 0.089), and defoliation depth (model weight: 0.088). Understory vegetation cover (model weight: 0.103), soil pH (model weight: 0.103), and size (model weight: 0.097) were among the best model predictors for estimating beetle activity density with ΔAICc ≤ 1.94.

Model selection on spider family richness estimates produced three candidate models. Only the predictors of weight of dried leaves (model weight: 0.206), soil pH (model weight: 0.166) and soil moisture (model weight: 0.080) fit the models (ΔAICc ≤ 1089); soil pH (model weight: 0.374) and soil moisture (model weight: 0.358) were the best predictors of spider activity density (ΔAICc ≤ 0.09).

### Association between community composition and in-situ environmental variables

**Figure 1:**
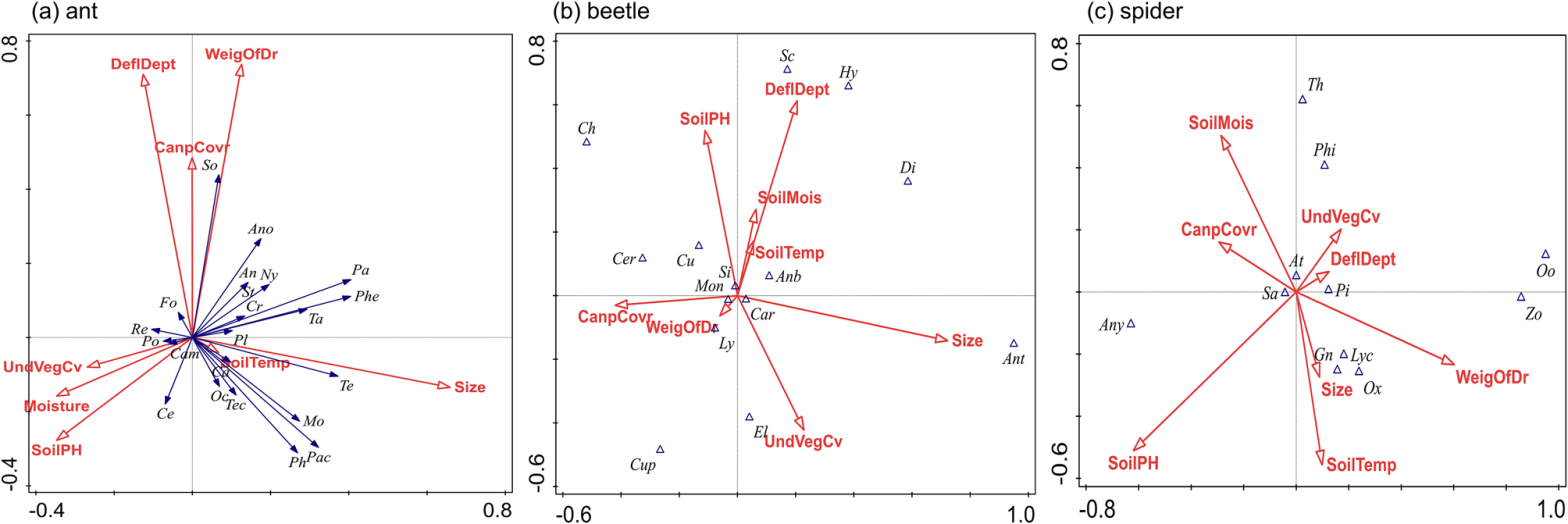
Redundancy analysis (RDA) biplot (**a**) showing the distribution of ant community composition (solid arrows) in relation to in-situ environmental variables (empty arrows). Canonical correlation analysis (CCA) biplot (**b**,**c**) showing the relationship between beetle and spider community (empty triangles) and in-situ environmental variables (empty arrows) in survey parks. Ant genera: Te, *Tetramorium*; Mo, *Monomorium*; Phe, *Pheidologeton*; Pa, *Paratrechina*; Ph, *Pheidole*; Ta, *Tapinoma*; Pac, *Pachycondyla*; Tec, *Technomyrmex*; So, *Solenopsis*; Cr, *Crematogaster*; Pl, *Plagiolepis*; Ca, *Cardiocondyla*; Ano, *Anoplolepis*; Oc, *Ochetellus*; Ce, *Cerapachys*; Cam, *Camponotus*; Po, *Polyrhachis*; Ny, *Nylanderia*; Fo, *Formica*; St, *Strumigenys*; An, *Anochetus*; Re, *Recurvidris*. Beetle families: *Car* carabidae; *Anb* anobiidae; *Ly* lymexylidae; *Si* silvanidae; *Cu* curculionidae; *Cer* ceratocanthidae; *El* elateridae; *Cup* cupedidae; *Ch* chrysomelidae; *Sc* scarabaeidae; *Hy* hybosoridae; *Ant* anthicidae; *Di* diphyllostomatidae; *Mon* monotomidae. Spider families: *Sa* salticidae; *At* atypidae; *Pi* pisauridae; *Lyc* lycosidae; *Ox* oxyopidae; *Oo* oonnopidae; *Any* anyphaenidae; *Gn* gnaphosidae; *Phi* philodromidae; *Th* thomisidae; *Zo* zodariidae. In-situ environmental variables: *Size* size of park; *CanpCovr* canopy cover; *UndVegCv* understory vegetation cover; *DeflDept* defoliation depth; *WeigOfDr* weight of dried leaves; *SoilTemp* soil temperature; *SoilMois* soil moisture; *SoilPH* soil pH.

The RDA for ant showed that the distribution of ant community along all axes was random (F-ratio = 1.1, *P* = 0.276, 999 permutations). The first two axes only explained approximately 14.32% of ant community composition. (Fig. 1a). For beetle and spider, similarly, CCA revealed that the community compositions distributed randomly along all axes (beetle: F-ratio = 1.1, *P* = 0.308, 999 permutations; spider: F-ratio = 0.9, *P* = 0.566, 999 permutations). 13.29% and 10.65% of the beetle and spider community compositions, respectively, were explained by the first two axes (Fig. 1b,c).

### The association between community structure, and park size and isolation

**Figure 2:**
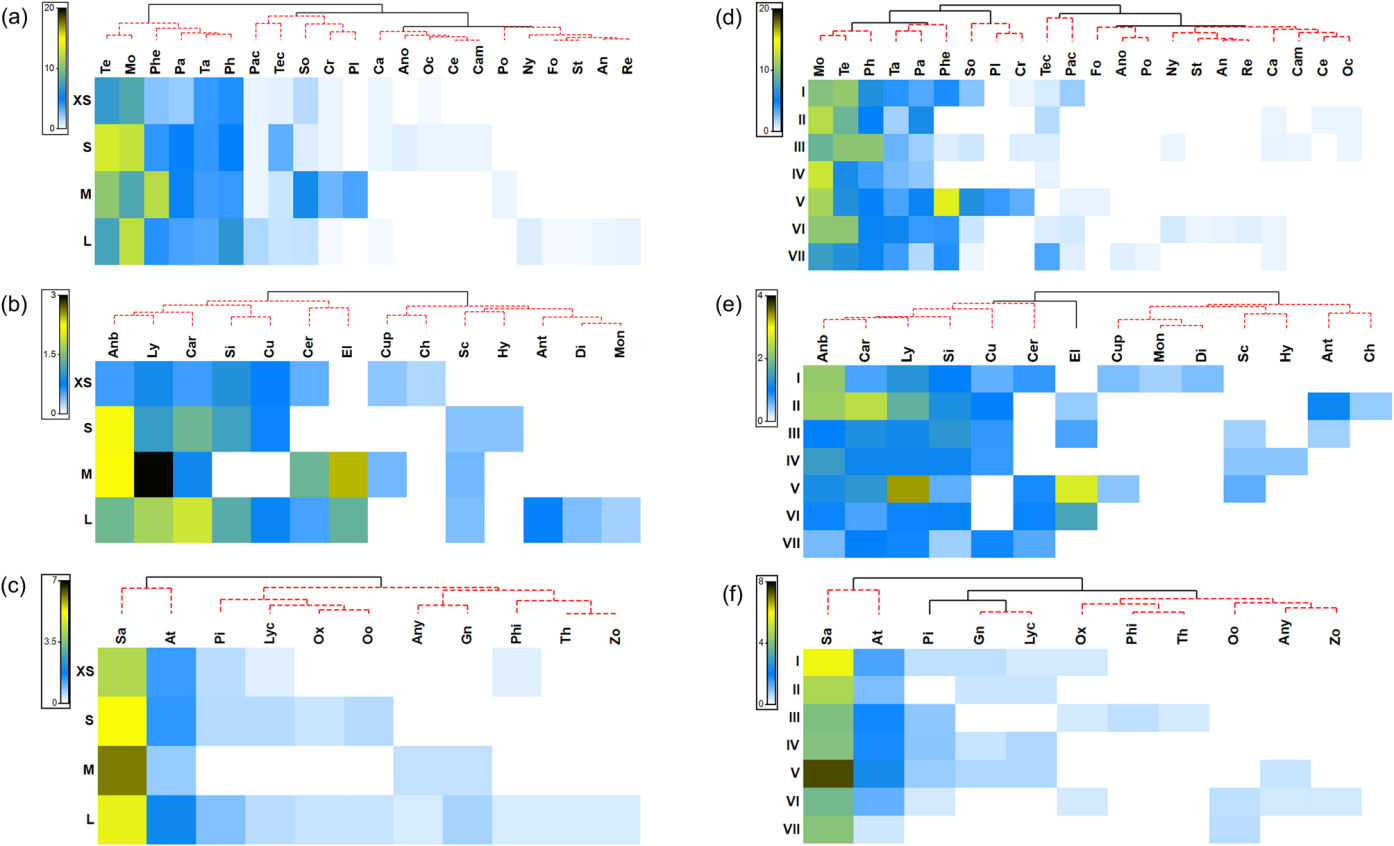
The similarity of community composition of ants (**a**,**d**), beetles (**b**,**e**), and spiders (**c**,**f**) in four park size groups (XS, extra small; S, small; M, medium; L, large) and seven isolation categories (I, < 1.0 km; II, 1.0–1.9 km; III, 2.0–2.9 km; IV, 3.0–3.9 km; V, 4.0–4.9 km; VI, 5.0–5.9 km; VII, > 6.0 km). Dendrograms of group-averaged clustering of test taxa are based on means of Bray–Curtis distance. Solid lines represent significant difference among groups based on SIMPROF (p < 0.05). Color scale represent mean abundance in each size of park. Abbreviations for ant genera: Te, *Tetramorium*; Mo, *Monomorium*; Phe, *Pheidologeton*; Pa, *Paratrechina*; Ph, *Pheidole*; Ta, *Tapinoma*; Pac, *Pachycondyla*; Tec, *Technomyrmex*; So, *Solenopsis*; Cr, *Crematogaster*; Pl, *Plagiolepis*; Ca, *Cardiocondyla*; Ano, *Anoplolepis*; Oc, *Ochetellus*; Ce, *Cerapachys*; Cam, *Camponotus*; Po, *Polyrhachis*; Ny, *Nylanderia*; Fo, *Formica*; St, *Strumigenys*; An, *Anochetus*; Re, *Recurvidris*; The beetle families are: *Car* carabidae; *Anb* anobiidae; *Ly* lymexylidae; *Si* silvanidae; *Cu* curculionidae; *Cer* ceratocanthidae; *El* elateridae; *Cup* cupedidae; *Ch* chrysomelidae; *Sc* scarabaeidae; *Hy* hybosoridae; *Ant* anthicidae; *Di* diphyllostomatidae; *Mon* monotomidae; The spider families are: *Sa* salticidae; *At* atypidae; *Pi* pisauridae; *Lyc* lycosidae; *Ox* oxyopidae; *Oo* oonnopidae; *Any* anyphaenidae; *Gn* gnaphosidae; *Phi* philodromidae; *Th* thomisidae; *Zo* zodariidae.

The SIMPROF revealed that the community structures of test taxa were significantly grouped (with 0.91, 0.82 and 0.91 of cophenetic correlation for ant, beetle and spider respectively) based on the taxa’s activity density in different sizes of parks (Fig. 2a–c). For instance, the ant genera *Nylanderia*, *Formica*, and *Strumigenys*; the beetle families Anthaicidae, Diphyllostomatidae, and Monotomidae; and the spider families Thomisidae and Zodariidae exclusively occurred in large parks (area of larger than 4 hectares). By contrast, the ant genera *Cardiocondyla* and *Ochetellus*, the beetle families Chrysomelidae and Hybosorisae, and the spider family Philodromidae were only present in small (area of 2.0–2.9 hectares) or extra small (area of less than 2.0 hectares) parks. However, the ant genera *Tetramorium*, *Monomorium,* and *Pheidologeton*; the beetle families Carabidae, Anobiidae, and Lymexylidae; and the spider families Salticidae and Atypidae were habitat generalists that occurred in all sizes of parks.

In terms of isolation, similarly, the community structures of test taxa based on the activity density were significantly grouped (with 0.91, 0.88 and 0.92 of cophenetic correlation for ant, beetle and spider respectively) in different categories (Fig. 2d–f). For instance, the ant genera *Camponotus*, *Cerapachys* and *Ochetellus*; the beetle families Monotomidae, Diphyllostomatidae and Anthicidae; the spider families Philodromidae and Thomisidae were occurred only in park that located less than 3 km from peri-urban forest. However, the ant genera *Strumigenys*, *Anochetus* and *Recurvidris*; and the spider families Oonopidae, Anyphaenidae and Zodariidae presented exclusively in parks that located more than 4 km from the nearby peri-urban forest. Moreover, the ant genera *Monomorium*, *Tetramorium* and *Pheidole*; the beetle families Anobiidae, Carabidae and Lymexylidae; and the spider families Salticidae and Atypidae were omnipresent at parks with varying distance categories.

## Discussion

Urban green areas are usually isolated and accompanied by impermeable surfaces, man-made structures, and elevated local temperatures^14^. Changes in habitat have a substantial effect on biodiversity and are inhospitable for environmentally sensitive species. Landscape and local effects can affect the species and relative abundance of different taxa. The results of the present study demonstrated a limited influx of individuals from source populations in the forests to adjacent urban parks except for beetle. However, some forest-dependent ants and spiders may utilize the surrounding matrix to migrate to distant parks. Species diversity of ants, beetles, and spiders increased with decrease of edge density in parks. In addition, these findings demonstrated that an increase in habitat heterogeneity engendered an increase in species diversity in large urban parks, which primarily supported the diverse community of urban-adapted species that responded favorably to the landscape change.

### Association between arthropod diversity and landscape configuration in urban parks

Contrary to our expectations, we did not identify a significant influence of distance from peri-urban forests in diversity metrics of ants and spiders. This nonsignificant association indicates no spillover effects from a source population to urban greeneries^13^. This result is consistent with those of studies conducted in less contrast silvicultural habitats^47,48^. However, studies of urban ground-dwelling ants in Rio de Janeiro City^49^ and two cities (Cordoba and Seville) of Southern Spain^50^ have reported an increase in species richness with increasing connectedness with forests. One of the proximate explanations concerning the absence of spillover effects in the present study is the size of the fragmented peri-urban forest, which is relatively small. The edge effect reduced the magnitude of the source population^47^. Nevertheless, we discounted the possibility that the small fragmented forests limited the spillover effects because the size of the peri-urban forest that we used as the representative of forest habitats is more than 11 500 ha. An ant inventory was established in the study sites and 60%–70% of ant assemblage identified in the forest borders were forest-dependent species^51^. Hogg and Daane^52^ reported that web-building spiders could disperse from oak woodlands to adjacent vineyards, whereas hunting spiders dispersed short distance within their original habitat (oak woodland) by ballooning with the aid of wind or rappelling from nearby trees. However, unlike juvenile spider, adult spiders do not generally disperse over long distances if a hunting site is ideal and will only launch less risky short-range dispersals^53,54^. In urban ecosystems, skyscrapers, buildings, and roads may become dispersal barriers leading to a limited spillover effect of the taxon from forest to urban parks as evidenced by the nonsignificant correlation between isolation and family diversity.

Despite the non-significant relationship in the present study, some rare forest-dependent ant genera such as *Pheidologeton*, *Polyrhachis, Pachycondyla* and *Recurvidris* were collected in urban greeneries located more than 5 km from the peri-urban forest. In the study, we did not sample any habitat other than urban parks (i.e., urban medians). However, the present result demonstrated that area of surrounding greenery had a clear positive influence in ant diversity metrices. Numerous studies have indicated that the urban matrix may contain intermediate to high ant species richness^55^, particularly in large medians^56^ and greenways^15^. Although ants are generally considered poor dispersers^57^, some individuals may utilize the nearest urban matrix as dispersal corridors to establish their colonies in distant urban parks. Similarly, Otoshi et al.^58^ demonstrated that the area of agricultural matrix within 1 km correlated with changes in spider activity density, particularly, the landscape effect was strongly positively correlated with adult spider and lycosid activity density. It is likely that urban parks may experience an influx of spider species from nearby agricultural matrix habitats, which generally attract high numbers and a variety of arthropods, through ballooning^58^.

Unlike ants and spiders, beetles are generally strong fliers and their colonization events from natural habitats to semi-natural landscapes within a short distance (less than 100 m spillover effects) were evident in areas of oil palm plantations adjacent to riparian reserve in Malaysia^47^ and grasslands adjacent to forest habitats in Italy^59^. This phenomenon was also observed in Germany^60^ in areas of crop field adjacent to seminatural habitats^61^. A meta-analyses of beetles in Europe, Japan and Canada demonstrated that the reduce in ground beetle assemblage may not necessary reflected in urbanized area because of the influx of non-forest species^62^. However, our empirical data did not support the hypothesis that the observed species enrichment of beetles in an urban landscape was caused by spillover effects alone. Instead, our results support a more complex interplay between peri-urban forest connectedness and edge density of a park. The latter effect similarly played a pronounced role affecting the diversity metrics of ants and spiders.

The proximate explanations regarding the negative association between diversity metrics and edge density of urban park are twofold. First, the edge effects may have a pronounced effect on species living in small urban greeneries compared with those living in large urban greeneries. The effect is particularly prominent in urban landscapes^63^ where urban development is always accompanied by intense road networks. Delgado, et al.^64^ studied the road edge effect on the temperature, light intensity, canopy cover, and tree height in laurel and pine forests in the Canary Islands and reported significant changes of temperature, light intensity, and tree structure from the road edge to the forest interior. A similar phenomenon was observed in our study sites; the rise in mean soil temperature was paralleled by an increase in park edge density (Appendix Table 1; Appendix Fig. 1). For a small patch size with an increased edge-to-interior ratio, the edge effects may reach the park interior and affect the survival of forest-dependent species. This hypothesis is supported by numerous empirical works on carabid beetle assemblages in urban greenspaces, which have reported that total species richness and abundance are, in general, lower in smaller patch area compared than in larger patch areas^65,66^. For instance, forest specialist species had a high affinity to inhabit large forest fragments with low edge density, whereas generalists tended to be observed in small forests^65^. We observed several beetle families exclusively inhabit large parks (area > 4 ha), whereas smaller parks (area < 4 ha) principally harbored habitat generalist species. Beetles that were exclusively observed in large parks required stringent local habitat requirements for survival, including more vegetation cover or prey availability at another location^14,67^.

Studies have drawn differing conclusions regarding ant responses to edge effects in urban landscapes. For instance, Clarke, et al.^68^ collected samples from 24 urban natural areas in San Francisco, California, and reported that natural area size and shape did not accurately predict ant species richness and abundance, with numerous smaller natural areas harboring diverse ant populations similar to larger areas. Studies in other semi-natural landscapes also demonstrated that the forest edge is an overlapping habitat of two habitat affinity groups, the forest specialist and open habitat species, and thus may harbor more ant^69^, beetle^70^, spider species^71^ as well as other arthropods^72^. However, in the present study, we determined that smaller parks may be subjected to larger edge effects and contain lower diversity. These results may be contradictory because of the variety of the surrounding habitat matrix in the study sites. For instance, principally positive effects were reported in forest–urban grass dominated habitats borders^69^, whereas, in the present study, park–pedestrian ways and asphalt roads produced heat radiation and contributed to unfavorable living environments in the neighboring habitats.

Second, another nonexclusive phenomenon that may result from the edge effects is the interspecific competition and ant displacement caused by urban-adapted ants, which may be more intense in smaller parks that experience fluctuating abiotic environmental conditions. The study sites contained five urban generalists, *Pheidole*, *Tetramorium*, *Monomorium*, *Tapinoma*, and *Paratrechina*, dominating the urban parks^12^. Their activity densities increased proportionally to the park size, indicating a proneness to habitat edge. These edge-prone ant species may have displaced forest-dependent ants and urban specialists. This hypothesis warrants further investigation. This hypothesis was corroborated by the invasive Argentine ant *Linepithema humile*, which thrives in moist edge habitats in natural boundaries and displaces native ants, beetles and spiders within at least 250 m from the urban edges^72–75^.

### Association between arthropod diversity and local habitat heterogeneity in urban parks

The diversity metrics of ants, beetles, and spiders increased with the increasing park size, reflecting a species–area relationship and indicating that the size of urban parks is critical for these arthropods. The results for ants were supported by a study conducted in New York City^56^, which indicated that ant species richness increased with increasing urban median areas. Similarly, a positive relationship between species number in a patch and the area of the patch of urban parks was also demonstrated in Tokyo metropolitan city and Chiba^76^. Moreover, MacGregor-Fors, et al.^77^ reported that the composition of copro-necrophagous beetles was related to the traits of greenspaces (size and location) in Xalapa, Mexico. However, these results contradict findings by Weller and Ganzhorn^6^ that species richness of carabid beetles decreased closer to the city center with an increasing degree of isolation of the sites but was uncorrelated with the size of the study sites. These asymmetric results may be because the study only investigated a single carabid beetle community, which only represented 34% occurrence of our sampling, and beetles from different family or feeding guilds may be more responsive to local effects^78^.

Instead we hypothesized that the increment was indirectly caused by its influence on habitat heterogeneity. We determined that the coefficient of variation of canopy openness, weight of dried leaves, and diameter at breast height of a standing trees increased with park size (Appendix Table 1; Appendix Fig. 2). Notably, ant genera such as *Paratrechina*, *Pheidologeton*, *Tapinoma*, *Tetramorium*, *Monomorium*, *Pachycondyla*, and *Pheidole* were tightly linked to park size. The ant genera *Solenopsis* and *Anoplolepis* may thrive in fine-scale heterogeneity across urban greeneries’ mosaics that have high plant litter mass and cool microhabitats. This may be because food sources are relatively rare and the environment warmer in urban areas compared with forests or woodlands. Therefore, environment-sensitive ants in an urban ecosystem are reliant on the dense ground cover for a food source. Microhabitats with less understory vegetation cover supported open habitat genera such as *Paratrechina* and *Tapinoma*, which are opportunists that usually inhabit dry and simple habitats and are characterized as poor competitors. These opportunist ant species are abundant when a given microhabitat contains low ant species number or less behaviorally dominant ants^79^. However, *Pheidologeton, Pheidole*, *Pachycondyla*, *Monomorium* and *Cerapachys*, which their occurrences were generally correlated with variables associated with forest, were present in relatively open areas in the present study^80^. We cannot explain precisely the reasons for this phenomenon but we should consider that such ant genera may have evolved to greater heat tolerance to occupy the vacant niche in order to escape interspecific competition.

We also identified a positive association between vegetation structure and beetle family richness and activity density. Vegetation was the most influential factor affecting beetle diversity^81^. In particular, the presence of vegetation provided an abundant food source for the understory herbivorous beetles’ (Hybosoridae and Scarabaeidae) survival and population growth^82^. Anobiidae, Lymexylidae, and Carabidae that represented the large proportion of the sampled population showed no compelling evidence for habitat preferences. Anobiid beetles and lymexylid beetles are bark/wood-boring pest that majorly found attacking wooden structures and living trees in urban areas^83^. Carabid beetles are generally active predators, less specialized and utilize a greater range of habitats whilst foraging. They are thermophilic and respond favorably to increases in ground temperature^84–86^. Rather than habitat complexity, carabid beetle occurrence may be more driven by the availability of food prey in urbanized landscapes. The results were somewhat opposite with the findings made by Lassau et al.^41^ that the habitat preference of anobiid beetles and carabid beetles were more associated with moist areas and habitats with high plant litter mass.

In spiders, we determined that microclimate factors, such as soil pH and temperature, and leaf litter displayed favorable fits in models, which accorded with findings by Argañaraz, et al.^87^ who evaluated urban green areas in Córdoba city, Argentina. This result is also consistent with that of Otoshi, et al.^58^ who determined that local fine-scale habitat quality (i.e., vegetation cover and species, bare soil) had a larger effect on the spider assemblage in 19 urban garden sites in three California counties (Santa Cruz, Santa Clara, and Monterey) compared with landscape-scale factors (i.e., gradient of development in surrounding areas). In opposite, Nagy et al.^78^ reported that no difference in diversities of spiders along the urbanization gradient in the city of Debrecen (Hungary) and its surrounding forested area.

Spiders, ants and carabid beetles are placed at a high trophic position among the test taxa in our study system. We determined that local resource availability may be an equally critical driving force in arthropod persistence in urban ecosystems^88^. Prey–predator dynamics have been widely studied in seminatural landscapes. However, similar patterns cannot be expected in urbanized areas, which are characterized by simplified landscape features. For instance, UHI effects play a pronounced role in one predator’s predation service. A study investigated how urban warming and herbivore abundance affected arthropod’s natural enemies in street trees and reported that the abundance of spiders did not increase linearly with herbivore abundance. This is because urban warming drastically simplified the community composition of spiders by diminishing the population of certain effective predators^89^. In the present study, Salticidae comprised 88.3% of the total sampled spiders, followed by Atypidae (8.4%). Salticid spiders are polyphagous predators that feed on a wide range of arthropods; the size of prey can reach twofold the size of the spider^90^. The prey–predator interactions have been revealed in various ecosystems^90–92^. Touyama, et al.^93^ sampled urban areas (e.g., urban parks and vacant lots) and reported that the frequency and density of the myrmecophagic jumping spider *Siler cupreus* Simon were significantly higher in sites infested with Argentine ants compared with infestation-free sites. Our unpublished data using stable isotope analysis revealed that ants in urbanized landscapes feed on a variety of insect. Information regarding the natural enemy abundance and diversity is insufficient to provide an overall picture of trophic interactions and predator efficiency of a species in a given ecosystem. A biological control in agricultural systems is harnessed by increased landscape complexity^94–98^, but an opposing pattern has been documented in urbanized landscape^99–101^. Future research should focus on the predator efficiency of natural enemy of whether prey abundance increase near the mean levels of natural enemy abundance (numerical response) or its predation rate (functional response) in a given urbanized landscape.

## Conclusion

The results of the present study demonstrated that both landscape and local effects are important in shaping the diversity metrics of ants, beetles, and spiders in urban parks. However, this result should be viewed with caution owing to a lack of high taxonomic resolution because even species within a family may have different tolerances to novel environments in urbanized landscapes. The present results indicated that man-made structures have been effective dispersal barriers that limit the spillover effects of ant and spider to urban park except for relatively strong flying beetles. However, urban greenery at the surrounding matrix potentially facilitates the colonization of the two poor dispersers in the distant parks. The edge density of a given urban park appears to be a major assembly rule in shaping the test taxa. Two proximate mechanisms of edge density effects are (1) an increased edge-to-interior ratio in small parks created hotter and drier areas relative to large parks, which is inhospitable to environment-sensitive taxa. (2) Arthropod displacement by edge-adapted ants. Local fine-scale heterogeneity also significantly explained some of the variability identified in different sizes of parks. In particular, ants, beetles, and spiders are sensitive to changes in the local vegetation structure, especially those in understory plant litter, which can provide shelter, hibernation sites, oviposition sites, and foraging sites for both predators and prey in urban parks^20,102^. We suggest that focusing on the local management of ground features in urban parks, regardless of park size, may be the optimal approach to maximize the conservation of generalist predators and harness their ecosystem services. Urban greeneries including urban parks are the hotspots for biodiversity in an urban environment and can accommodate over 50% of the species present in peri-urban areas. The richness could be further enhanced if effective management is implemented^103^. Those urban-adapted ground-dwelling insects are keystone species and may mediate the local biological interactions and shape arthropod assembly in urban greeneries.

## Supplementary information

Supplementary Information.
