# Supplementary material for: Landscape configuration and habitat complexity shape arthropod assemblage in urban parks
Source: Sci Rep. 2020 Sep 29;10:16043. doi: 10.1038/s41598-020-73121-0 (PMC7525568; doi:10.1038/s41598-020-73121-0)
Supplement: Supplementary file 1 — Supplementary Information. [file 41598_2020_73121_MOESM1_ESM.docx]

For: Scientific Reports

Corresponding author:

Kok-Boon Neoh

Department of Entomology

National Chung Hsing University

145 Xingda Rd.,

Taichung, 402 Taiwan

Tel. no.:+886(04)22840361

Fax: +886(04)22875024

Peng et al.: Arthropod assemblages in urban parks

**Landscape configuration and habitat complexity shape arthropod assemblage in urban parks**

Ming-Hsiao Peng^#^, Yuan-Chen Hung^#^, Kuan-Ling Liu, Kok-Boon Neoh^*^

Department of Entomology, National Chung Hsing University, 145, Xingda Rd. South District, Taichung 402 Taiwan

# denotes equal contribution

^*^ Corresponding author: Email: neohkokboon@yahoo.com

Appendix 1

We were interested, to what extent, the size of park affected soil temperature, soil moisture, and coefficient of variation (C.V) of in-situ environment parameters (e.g. canopy cover, understory vegetation cover, weight of dried leaves and diameter at breast height of a standing tree). Generalized linear mixed-effects models (GLMMs) were used to examine the effect of park size on the environmental variables using a Gaussian error distribution. Surveyed parks were considered the random effects, the park size as a fixed effect, and environmental variables as the response variables. The GLMM analysis was performed using R version 3.4.1 and the lme4 version 1.1-13 package.

Appendix Table 1. Results of the GLMM testing response of in-situ environmental variables to size of park.

| Response variables | Estimation | Standard error | Statistical value | Conditional R^2^ |
| --- | --- | --- | --- | --- |
| Soil temperature (⁰C) | -0.607 | 0.410 | -1.480 | 0.882 |
| Soil moisture (%) | 0.004 | 0.013 | 0.301 | 0.877 |
| C.V of canopy cover | 0.215 | 0.092 | 2.328 | 0.902 |
| C.V of understory vegetation cover | -0.307 | 0.218 | -1.407 | 0.887 |
| C.V of weight of dried leaves | 0.002 | 0.226 | 0.009 | 0.877 |
| C.V of diameter at breast height of a standing tree | 0.073 | 0.072 | 1.019 | 0.883 |


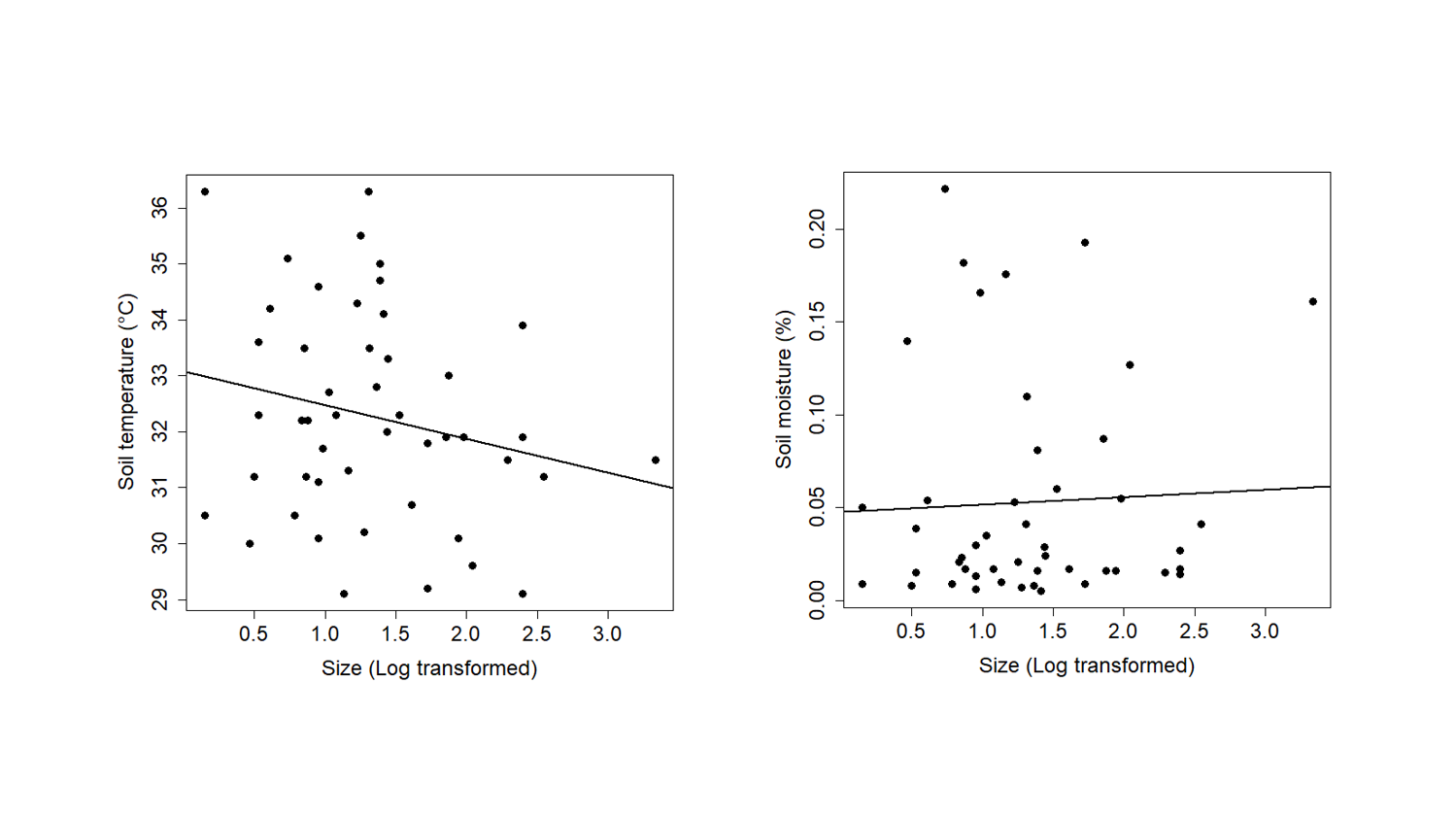


Appendix Fig. 1. The relationship between soil temperature (⁰C) and soil moisture (%), and size of park.


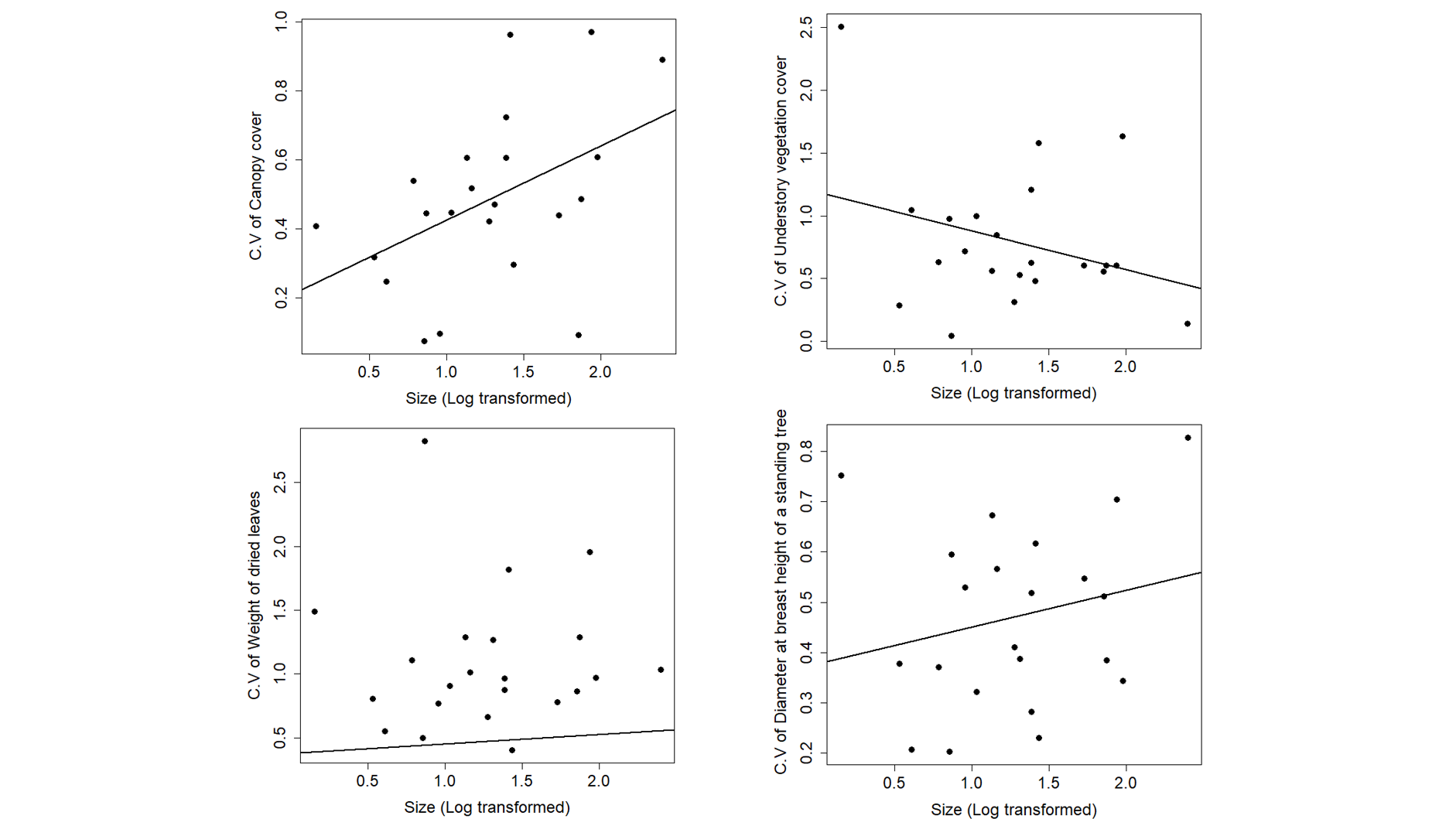


Appendix Fig. 2. The relationship between coefficient of variation of canopy cover, understory vegetation cover, weight of dried leaves and diameter at breast height of a standing tree, and size of park.
